# Multimodal imaging of Spina Ventosa (TB Dactylitis) of the foot

**DOI:** 10.1016/j.radcr.2020.05.027

**Published:** 2020-06-27

**Authors:** Mark C. Murphy, Alexandra N. Murphy, Hannah Hughes, Owen J. McEneaney, Conor O'Keane, Eoin Kavanagh

**Affiliations:** a Department of Radiology, Mater Misericordiae University Hospital (University College Dublin), Eccles St, Dublin 7, Dublin, Ireland; b Department of Orthopaedic Surgery, Mater Misericordiae University Hospital (University College Dublin), Eccles St, Dublin 7, Dublin, Ireland; c Department of Pathology, Mater Misericordiae University Hospital (University College Dublin), Eccles St, Dublin 7, Dublin, Ireland

**Keywords:** TB dactylitis, Spina Ventosa, Osteomyelitis

## Abstract

We present the case of a 29-year-old male healthcare worker with a 6 month history of progressive left foot pain resulting in presentation to the emergency department on 3 occasions. He denied systemic symptoms. Multimodal imaging demonstrated an expansile erosive inflammatory lesion centered on the neck of the second metatarsal with aggressive features. CT of the thorax, abdomen, and pelvis demonstrated calcified mediastinal lymph nodes and left inguinal adenopathy. The lesion was biopsied under ultrasound guidance demonstrating a necrotizing granulomatous osteomyelitis with acid fact bacilli. This is consistent with TB dactylitis (spina ventosa). Treatment with antimycobacterial drugs was commenced.

## Case

**Fig. 1:**
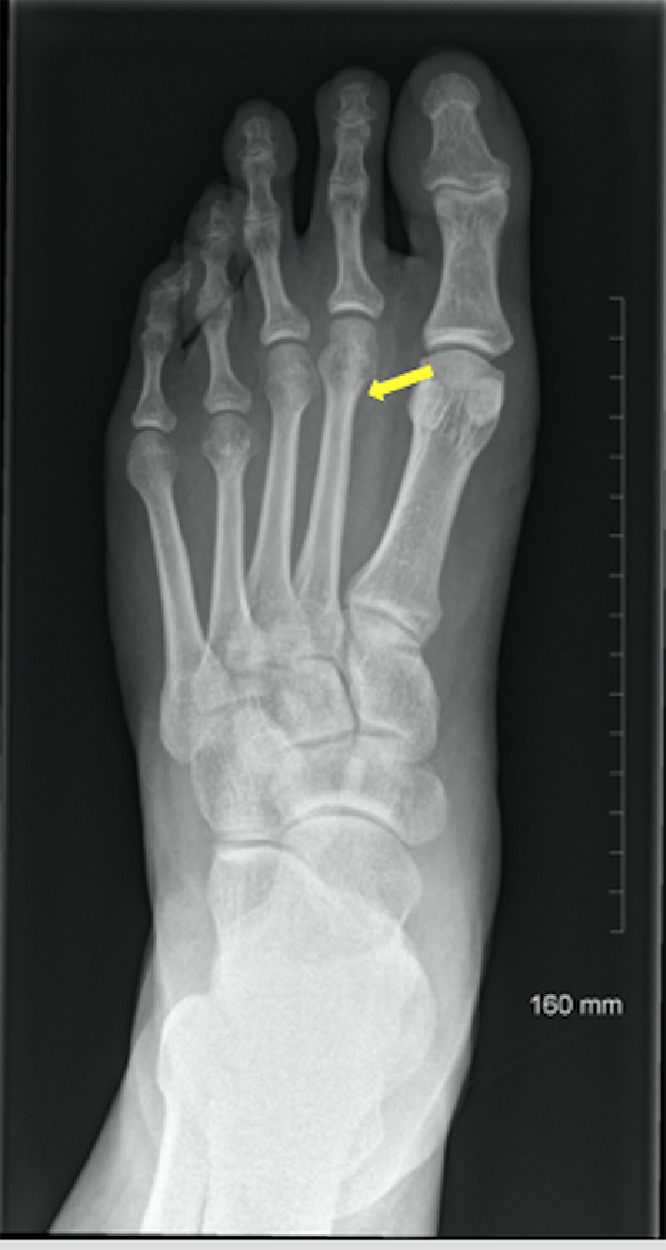
Oblique and DP radiographs of the left foot from Aug 2019, one month post initial presentation. There is very subtle periosteal reaction at the medial aspect of the neck of the second metatarsal (arrows).

**Fig. 2:**
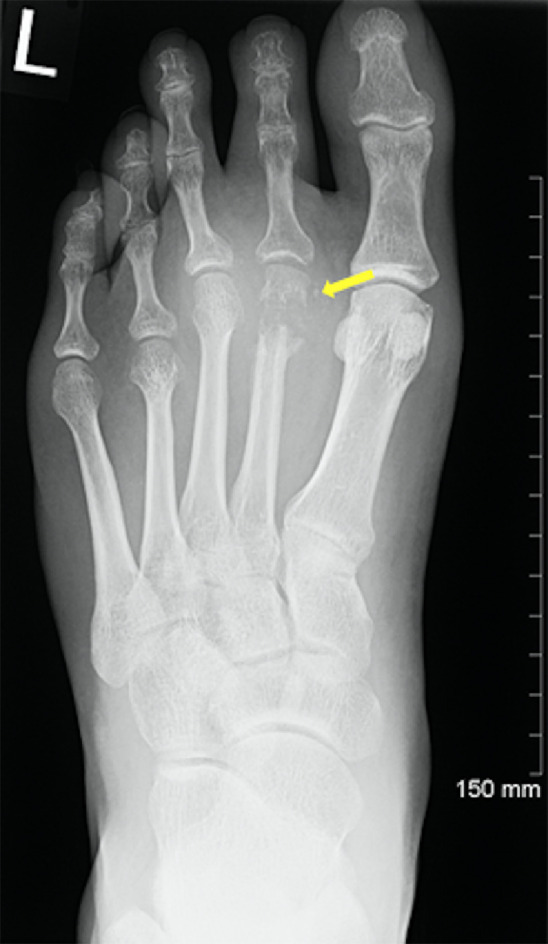
Oblique and DP radiographs of the left foot from Feb 2020, 6 months post initial presentation. There is marked expansile metaphyseal and cortical erosion centered on the neck of the second metatarsal (arrows). There is associated soft tissue swelling with some internal calcification.

**Fig. 3:**
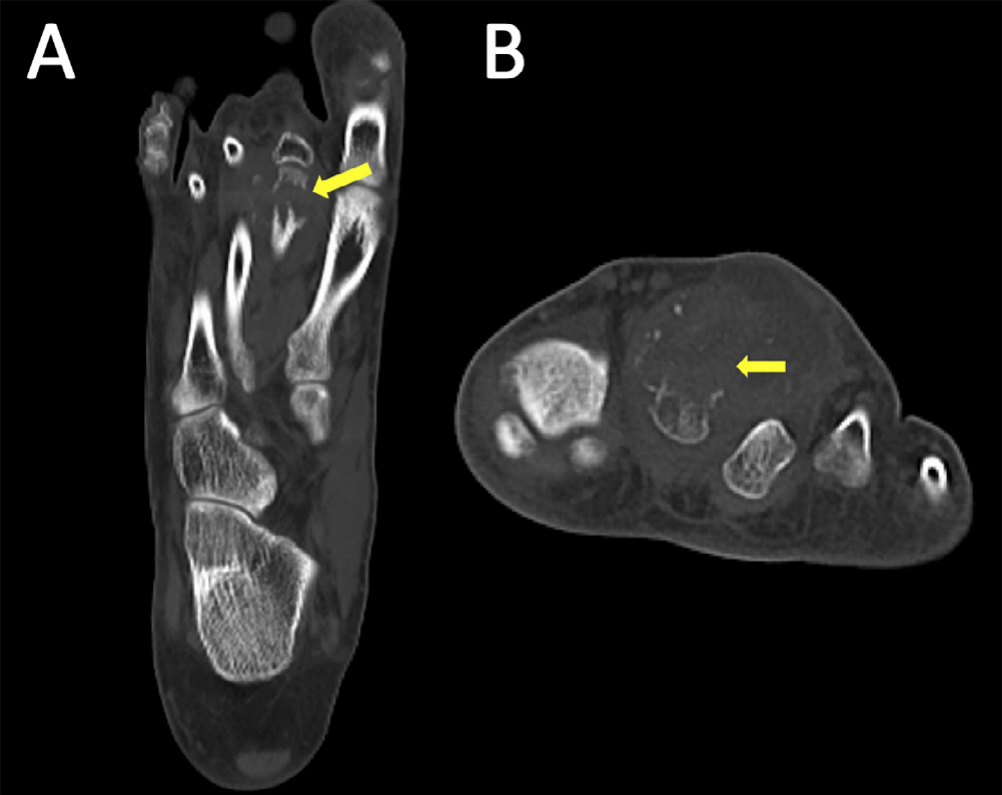
Long axis (A) and short axis (B) Cone beam CT images of the left foot. There is an expansile soft tissue lesion centered on the second metatarsal neck (arrows). There are several foci of calcification within the lesion, similar in appearance to a chondroid matrix.

**Fig. 4:**
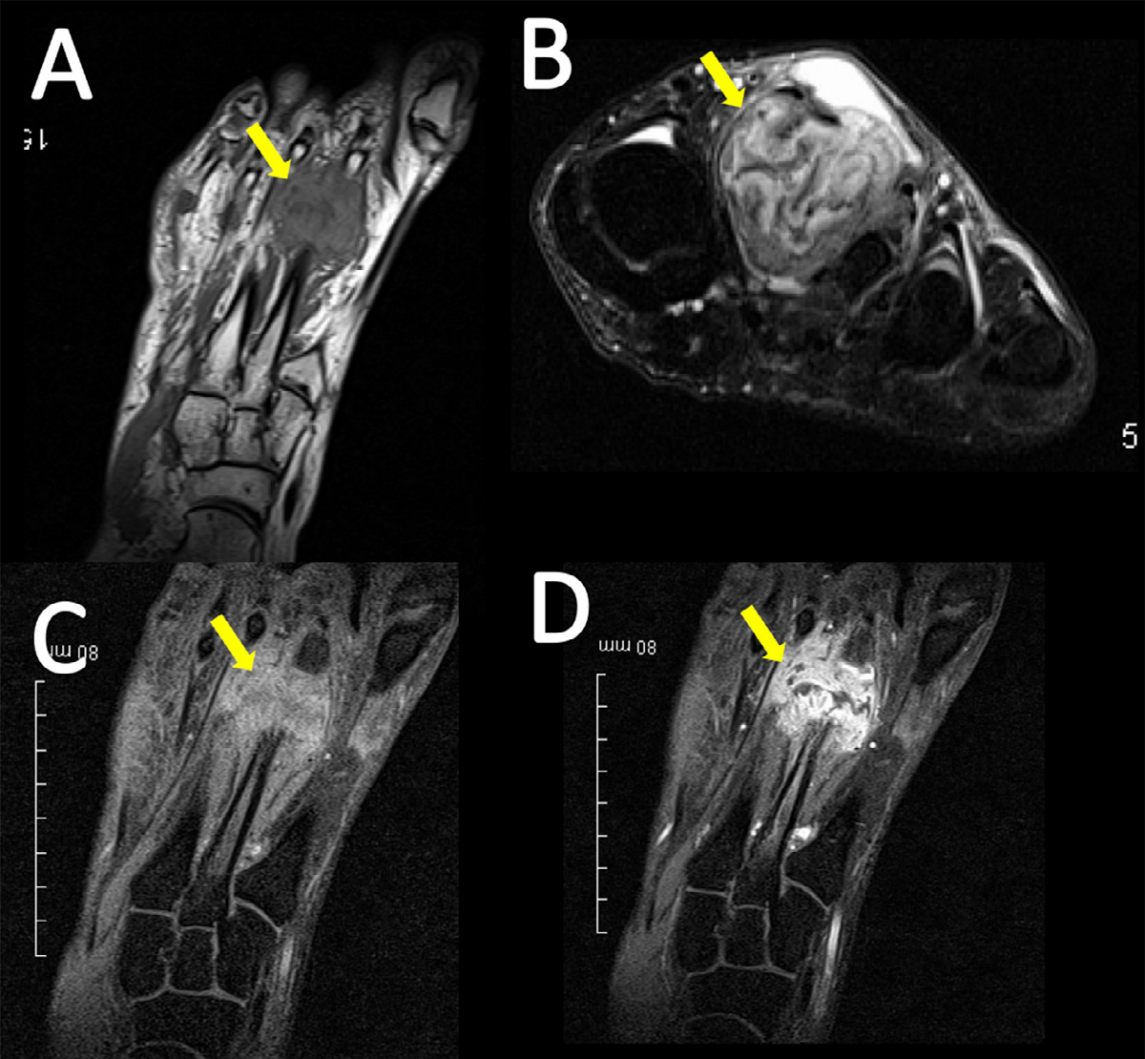
(A) Long axis T1, (B) short axis T2 STIR, (C) long access T1 STIR Pre contrast, (D) long axis T1 STIR post contrast MRI sequences of the left foot. The expansile soft tissue lesion (arrows) demonstrates T1 low signal with T2 heterogenous high signal. There is a large effusion at the dorsal aspect. There is avid post contrast enhancement within the lesion and further enhancement within the marrow of the second metatarsal. Punctate areas of signal drop out correspond to foci of calcification on CT.

**Fig. 5:**
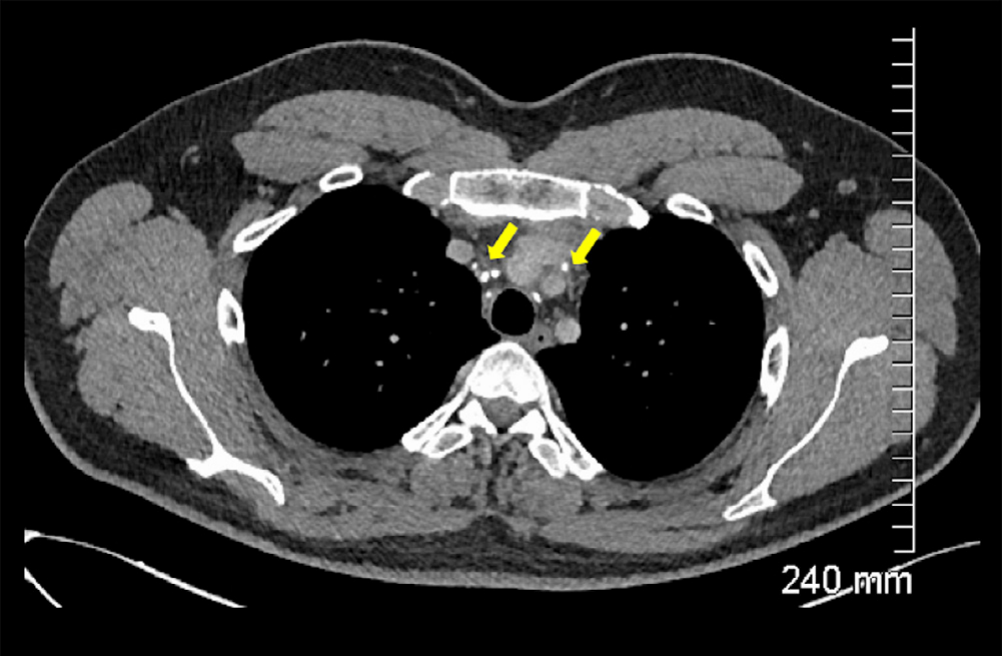
Axial contrast enhanced CT of the thorax demonstrating calcified small mediastinal lymph nodes (arrows).

**Fig. 6:**
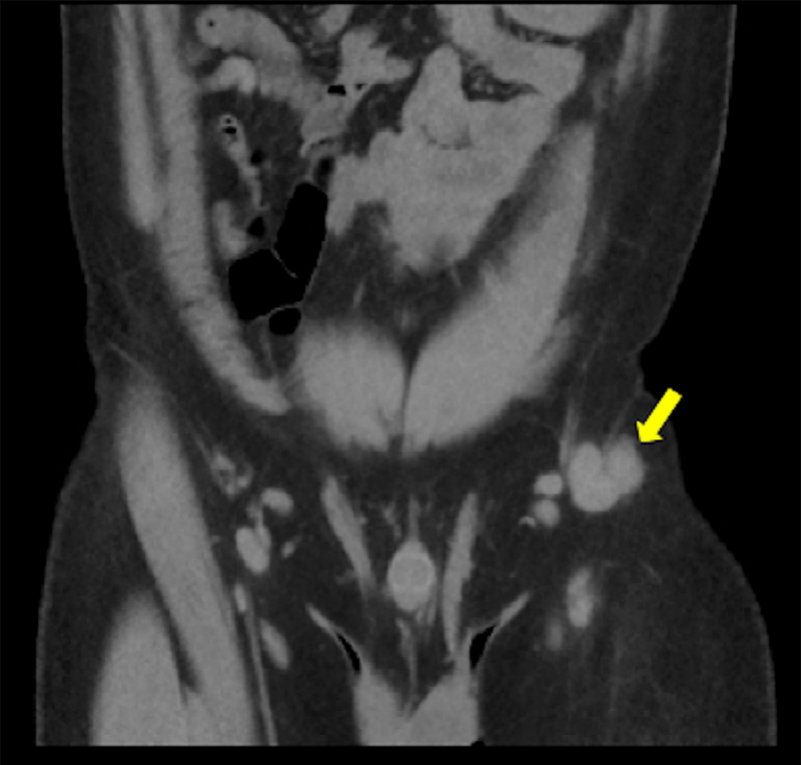
Coronal contrast enhanced CT of the pelvis demonstrating enlarged left inguinal lymph nodes (arrow).

**Fig. 7:**
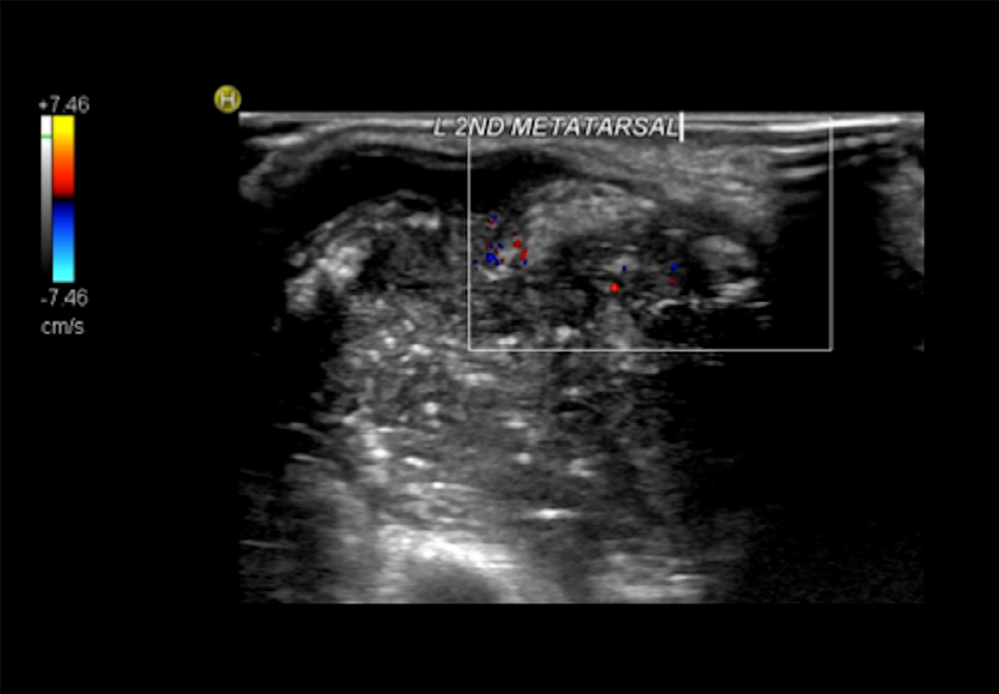
Transverse color Doppler ultrasound of the left foot focused on the soft tissue mass at the neck of the second metatarsal demonstrating increased vascularity at the periphery of the lesion.

**Fig. 8:**
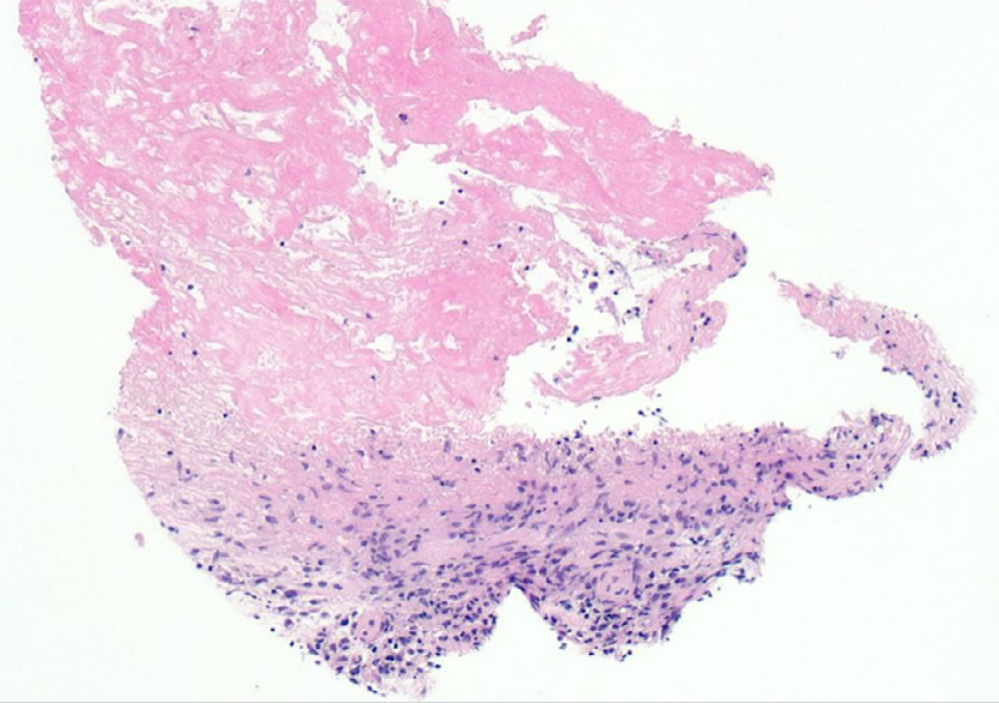
Histology slide with H&E stain 100x magnification demonstrating necrotizing granulomatous inflammation.

**Fig. 9:**
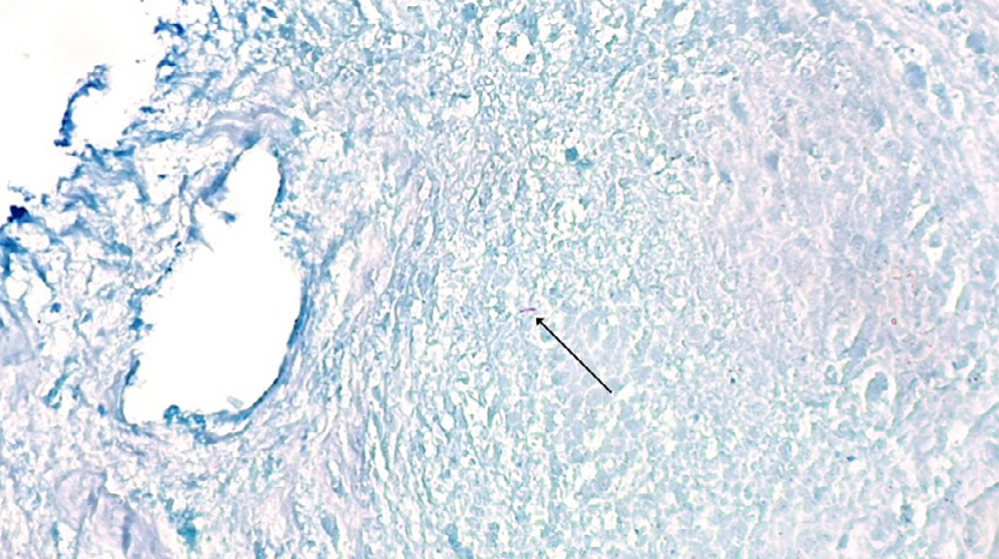
Histology slide with ZN stain at 400x magnification demonstrating a single acid fast bacillus within necro-inflammatory debris.

**Fig. 10:**
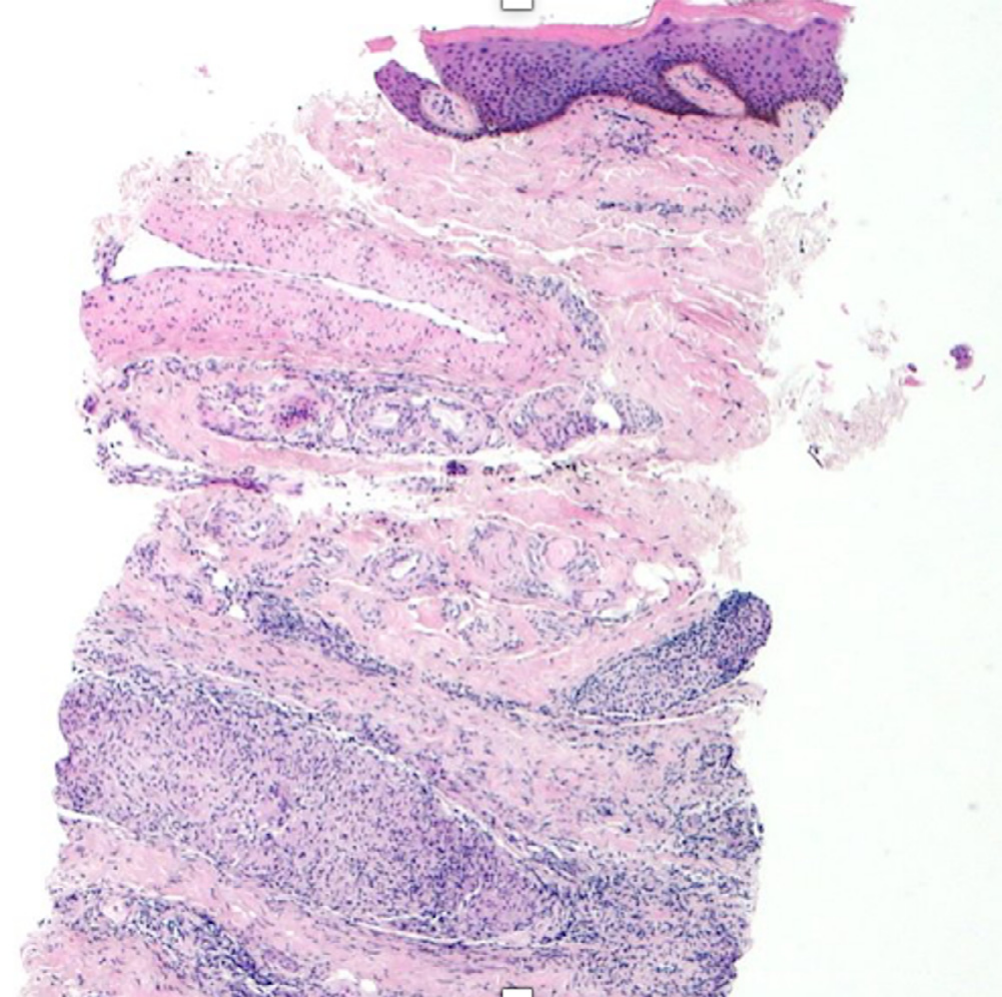
Histology slide with H&E stain at 40x magnification demonstrating granulomatous inflammation in overlying soft tissue/skin.

A 29-year-old male presented to the emergency department in July 2019 with an episode of left foot pain localized to the second/third metatarsal heads, first noticed after playing football. The patient was originally from the Indian subcontinent, living and working as a healthcare worker for several years. Left foot radiograph was normal. He represented one month later and a repeat radiograph was performed which demonstrated subtle periosteal reaction at the neck of the 2nd metatarsal (Fig. 1). He was treated as a stress fracture and managed conservatively. The patient represented 6 months later with persistent non-resolving pain. He was fully weightbearing on the foot and denied systemic symptoms such as fever or night-sweats. Physical examination demonstrated a firm mass over the forefoot with no pain on palpation. A repeat radiograph demonstrated an expansile lesion at the distal diaphysis of the second metatarsal with extensive periosteal erosion Fig. 2. Urgent outpatient CT and MRI of the foot were performed. CT confirmed the presence of an expansile lesion centered on the second metatarsal neck Fig. 3. Several foci of increased attenuation within the lesion were suggestive of an internal chondroid matrix. On MRI the lesion demonstrated low signal on T1 and heterogenous increased signal on T2 weighted sequences. There was avid enhancement within the lesion and the marrow of the second metatarsal diaphysis (Fig. 4). Overall imaging appearances were concerning for a primary bone neoplasm with chondrosarcoma the favored differential. The patient underwent staging CT thorax, abdomen, and pelvis which demonstrated some calcified mediastinal lymph nodes (Fig. 5) and further prominent left inguinal nodes (Fig. 6). The lungs were clear. The patient then underwent ultrasound guided biopsy (Fig. 7) and the sample was sent to histology. Histology demonstrated necrotizing granulomatous inflammation with a single acid fast bacillus within the necrotizing debris (Fig. 8, Fig. 9, Fig. 10). This is consistent with mycobacterium tuberculosis.

## Discussion

Tuberculous dactylitis is a rare manifestation of primary tuberculous infection involving the short tubular bones of the hand or feet [1]. The aetiology is thought to be haematogenous spread of infection [2]. In the early course of the disease there is soft tissue swelling and periostitis. As the disease progresses there is expansile bone destruction and The diagnosis of spina ventosa is made when there is expansile bony destruction and formation of a sequestrum, a segment of necrotic bone [3]. The name is derived from the Latin words “spina” meaning short bones, and “ventosa” meaning expanded with air [4]. Over 85% of cases affect children [5] with the hands being more commonly affected than the feet. In children the condition usually affects multiple bones whereas in adults a single site of disease is more common [4,5]. The radiologic differential diagnosis includes primary bone tumors, pyogenic or fungal infections, syphilitic dactylitis, sarcoidosis, hemoglobinopathies, hyperparathyroidism, and leukemia [3]. Unlike acute pyogenic osteomyelitis, patients with tuberculous dactylitis usually experience a more indolent course, rarely manifesting systemic symptoms [6].

This adult case of tuberculous dactylitis of the foot represents a rare manifestation of a rare disease. The case highlights the indolent nature of the infection with initial imaging normal or very subtly abnormal. The expansile bony destruction took 6 months to manifest. Multimodal imaging appearances are non specific and mimic more aggressive processes such as primary bone neoplasms. The clinical presentation is also very non specific. For this reason multidisciplinary input is required.
